# Building a house without foundations? A 24-country qualitative interview study on artificial intelligence in intensive care medicine

**DOI:** 10.1136/bmjhci-2024-101052

**Published:** 2024-04-19

**Authors:** Stuart McLennan, Amelia Fiske, Leo Anthony Celi

**Affiliations:** 1 Institute of History and Ethics in Medicine, Department of Preclinical Medicine, TUM School of Medicine and Health, Technical University of Munich, Munich, Bavaria, Germany; 2 Institute for Biomedical Ethics, University of Basel, Basel, Switzerland; 3 Laboratory for Computational Physiology, Massachusetts Institute of Technology, Cambridge, MA 02139, USA; 4 Division of Pulmonary, Critical Care and Sleep Medicine, Beth Israel Deaconess Medical Center, Boston, MA 02215, USA; 5 Department of Biostatistics, Harvard T.H. Chan School of Public Health, Boston, MA 02115, USA

**Keywords:** Information Science, Artificial intelligence

## Abstract

**Objectives:**

To explore the views of intensive care professionals in high-income countries (HICs) and lower-to-middle-income countries (LMICs) regarding the use and implementation of artificial intelligence (AI) technologies in intensive care units (ICUs).

**Methods:**

Individual semi-structured qualitative interviews were conducted between December 2021 and August 2022 with 59 intensive care professionals from 24 countries. Transcripts were analysed using conventional content analysis.

**Results:**

Participants had generally positive views about the potential use of AI in ICUs but also reported some well-known concerns about the use of AI in clinical practice and important technical and non-technical barriers to the implementation of AI. Important differences existed between ICUs regarding their current readiness to implement AI. However, these differences were not primarily between HICs and LMICs, but between a small number of ICUs in large tertiary hospitals in HICs, which were reported to have the necessary digital infrastructure for AI, and nearly all other ICUs in both HICs and LMICs, which were reported to neither have the technical capability to capture the necessary data or use AI, nor the staff with the right knowledge and skills to use the technology.

**Conclusion:**

Pouring massive amounts of resources into developing AI without first building the necessary digital infrastructure foundation needed for AI is unethical. Real-world implementation and routine use of AI in the vast majority of ICUs in both HICs and LMICs included in our study is unlikely to occur any time soon. ICUs should not be using AI until certain preconditions are met.

**WHAT IS ALREADY KNOWN ON THIS TOPIC**

- Existing research on intensive care professionals’ views about artificial intelligence remains limited and only includes participants from four high-income countries.

**WHAT THIS STUDY ADDS**

- This is one of the largest qualitative studies to date to examine the views of intensive care professionals regarding the use and implementation of AI technologies in intensive care units, involving 59 participants from 24 countries. It shows that the vast majority of ICUs neither have the technical capability to capture the necessary data or run AI algorithms, nor the staff with the right knowledge and skills to use the technology as designed.

**HOW THIS STUDY MIGHT AFFECT RESEARCH, PRACTICE OR POLICY**

- Pouring massive amounts of resources into developing AI without first building the necessary digital and knowledge infrastructure foundation needed for AI is unethical and needs to change.

## Introduction

Intensive care medicine has long been at the forefront of efforts to use routinely collected digital health data to improve patient care,^1–3^ and it is seen to be particularly well positioned to use the advances in artificial intelligence (AI) given the amount of data typically generated in intensive care units (ICUs).^4^ It is expected that applications of AI in ICUs will primarily be focused on machine learning to assist in disease identification, prediction of disease progression, disease phenotyping, recognising unique patterns within complex data and guiding clinical decision-making.^5–7^ Other potential applications include algorithms taking a physically embodied presence, such as in smart autonomous ventilators or infusion pumps.^8 9^ Despite the anticipated benefits AI technology, a large ‘implementation gap’ between what has been developed and what is used in clinical practice continues to grow, with most developed ICU AI models remaining in testing and prototyping.^10 11^ Challenges for the successful development and implementation of AI tools in ICUs have been increasingly researched and discussed in recent years,^4–17^ including: (1) various technological challenges around obtaining high-quality data; ICU data is often heterogeneous and noise-prone, and de-identifying, standardising, cleaning and structuring the data can be difficult^4–6 11 14^; (2) a number of general ethical, legal and regulatory issues, particularly around data protection and sharing^5 6 18^; (3) the vast majority of ICU AI models are not robust or ready for clinical use; they have been developed using retrospective data, without external validation or prospective evaluation^6 15^; and (4) obtaining the trust and acceptance of clinicians and other stakeholders.^5 6^ Indeed, it is important to better understand intensive care professionals’ views and acceptance of AI to help identify key barriers and facilitators to AI technology being implemented and adding value to intensive care medicine. At the time of designing and initiating this study, there was a lack of empirical studies on intensive care professionals’ views about AI. However, in the past 2 years, a few quantitative and qualitative studies have been published.^13 14 19 20^ These studies have found general positive attitudes and expectations of ICU professionals towards the use of AI, but also primarily identified technical barriers to the implementation of AI in ICUs. In addition, they identified some non-technical factors (a lack of AI knowledge among ICU professionals, high clinical workload, no clear AI policy, a lack of funding for digitalisation and a culture of doctor-knows-best). However, these studies have consisted of three small survey studies involving one centre^13 20^ or two centres^19^ from the Netherlands or the USA, and an interview study including participants from the USA and three European countries (the Netherlands, Belgium and the UK).^14^ Existing research on intensive care professionals’ views about AI therefore remains limited and only includes participants from four high-income countries (HICs). Furthermore, HICs have so far dominated the discussion over AI and related ethical issues.^21^ In an era of increasing global collaborative health research efforts, this imbalance is problematic. Lower-to-middle-income countries (LMICs) are also increasingly using healthcare data science and AI.^22–25^ This study therefore aims to explore the views of intensive care professionals in both HICs and LMICs regarding the use and implementation of AI technologies in ICUs.

## Methods

This study is presented in accordance with the Consolidated Criteria for Reporting Qualitative Research reporting guideline.^26^ See online supplemental information 1 for additional details on methods used in the study. Intensive care professionals were primarily selected through purposive sampling to ensure that participants were from different backgrounds and regions.^27^ The classification of a country as an HIC or an LMIC was taken from the Statistical Annex of the World Economic Situation and Prospects 2022.^28^ Additional participants were identified using snowball sampling.^29^ 59 intensive care professionals (physicians, nurses, pharmacists, physical therapists) from 24 countries agreed to participate. Interviews were held via telephone or video call between December 2021 and August 2022. All interviews were conducted in English, except for seven interviews which were held in Spanish. A researcher-developed semi-structured interview guide was developed to guide the discussion (see online supplemental information 2). It should be noted that the interviews were conducted prior to the release of ChatGPT and other chatbots powered by large language models (LLMs).^30^ Interviews were audio recorded and transcribed and were analysed in their original language using conventional content analysis with the assistance of the qualitative software MAXQDA (VERBI Software).^31^

10.1136/bmjhci-2024-101052.supp1 Supplementary data

10.1136/bmjhci-2024-101052.supp2 Supplementary data

## Results

Among the 59 intensive care professionals who participated in the study, 69.5% were physicians (41/59), 18.6% were nurses (11/59), 6.8% were pharmacists (4/59) and 3.4% were physical therapists (2/59). Overall, 23.7% of participants were from Europe (14/59), 16.9% were from Asia (10/59), 15.3% were from North America (9/59), 13.6% were from South America (8/59), 11.9% were from the Middle East (7/59), 10.2% were from Australasia (6/59) and 6.8% were from sub-Saharan Africa (4/59). Furthermore, 66.1% (39/59) of participants were male presenting (table 1).

**Table 1:** Participants demographics

Characteristic	Total
Gender	
Male	39/59 (66.1)
Female	20/59 (33.9)
Position	
ICU physicians	41/59 (69.5)
ICU nurses	11/59 (18.6)
ICU pharmacist	4/59 (6.8)
ICU therapist	2/59 (3.4)
Other	1/59 (1.7)
Region	
Europe	**14/59** (**23.7**)
Germany	3
France	2
Switzerland	1
Spain	1
The Netherlands	4
UK	4
Asia	**10/59** (**16.9**)
China	2
Hong Kong	2
India	2
Japan	2
Philippines	2
North America	**9/59** (**15.3**)
Canada	3
USA	5
Mexico	1
South America	**8/59** (**13.6**)
Argentina	3
Columbia	5
Middle East	**7/59** (**11.9**)
Qatar	4
Israel	2
Jordan	1
Australasia	**6/59** (**10.2**)
Australia	4
New Zealand	2
Sub-Saharan Africa	**4/59** (**6.8**)
Botswana	2
Malawi	1
Rwanda	1

ICU, intensive care unit.

### Status quo—patient data collection, documentation and utilisation

Most participants described a pervasive lack of digital data collection and documentation, and a chronic underutilisation of patient data in ICUs in both HICs and LMICs. In relation to patient data collection and documentation, most ICUs were reported to be paper-based or partially digitalised. Although patient data may be being collected with electronic monitors in these ICUs, it is typically documented manually either in paper-based records or in electronic health records. Consequently, the amount of available digital data was reported to be limited in most ICUs. With regard to the use of patient data for purposes other than patient care, although most ICUs are using data for national quality benchmarking data sets, the secondary use of patient data was reported to be extremely limited or non-existent by most participants.

Only a few participants working in a small number of large tertiary hospitals in HICs reported that patient data in their ICUs were primarily being automatically collected and documented digitally and being extensively used for secondary purposes. However, these were outliers and participants reporting that most other ICUs within the same country or even city as these fully digitalised ICUs were only paper-based or partially digitalised. Furthermore, even in most fully digitalised ICUs, it was reported that data is still required to be manually verified at regular intervals due to regulatory requirements to ensure data validity. Nevertheless, participants noted that in practice large amounts of data would often be confirmed without detailed verification. A minority of participants reported that verification is not required in their ICU; they want the raw data and did not think that nurses at the bedside were best placed to check data validity and that their time would be best spent on other tasks (table 2).

**Table 2:** Status quo—patient data collection, documentation and utilisation

Theme		
**Code**	**Subcode**	**Example quote**
Data collection and documentation		
Implementation stage	Paper-based	‘We collect it manually. So, we have an admission book. So, when a patient come, we collect the personal information of the patient, there is like the name of the patient, where the patient is coming from…But then, on our daily monitoring…we have now the observation where we record all the vitals signs…we do that in the admission book, as well as the patient files. We do it manually. We don’t have like, electronic documentation.’ P2 ICU Nurse LMIC ‘Most of the systems in [Country] are still paper based. Certainly, in the ICU, we are probably well, 10 years behind our [Country] cousins and probably 15, 20 years behind the US in terms of the way that we manage data.’ P47 ICU Physician HIC
	Partial digitalisation	‘But the way that is transferred, there is that all the information is stored in the monitor, for example, or in a ventilator. So, it’s in there. You won’t lose. It’s in there. But then you have to go write down the numbers and then move to the computer and transfer those numbers in there. So, for us, that’s the big limitation. Because first, you cannot do it minute by minute. And second, it’s very time consuming for a person to transfer that. And third, you are not sure that the number that she’s transferring is a real number.’ P5 ICU Physician LMIC ‘Okay. So, we have an electronic medical record…It’s introduced manually. So, we don’t really have like an automatic process where the data is stored. So, basically doctors and nurses put the data in the Electronic Medical Record. So, that’s the way we have to restore information.’ P18 ICU Physician LMIC
	Full digitalisation	‘Yeah, 95% of it is now electronic. So, starting from the vital signs, these are imported through-, collected through a central monitor, which is monitoring every single patient bed. And from that central monitor, it goes into a centralized database. And we’re using the [Company name] system. And it’s recording minute by minute data. But for verification, and the nurse would chart the data every hour. And if there’s an event, which requires more frequent charting, for example, patients deteriorating or some sudden event, the nurse can then chart more data between hours. In terms of the lab data, it is collected via the hospital electronic database. So, our database goes and fetches data from the hospital database…So, we have to use-, we have to juggle a few systems at one time.’ P3 ICU Physician HIC ‘So, I can say confidently at this point, it’s 100% electronic documentation as far as vital signs goes. We have a, like a background software that transports patient’s vital signs, for ICU patients almost minute-by-minute to like a secondary software that we have that’s called [Name)…So, all the vital signs get automatically transcribed. Labs usually gets also documented from electronic medical records, also to that software. So, they're all on the same place. The-, like all the drip rates are manually entered by the nurses when they’re started, ended, up titrated or down titrated. All the nursing assessment…they all go in there by manual entry.’ P6 ICU Pharmacist HIC
	Variations within countries and regions	‘So, I’ve experienced a really wide variation….I’ve worked in four different hospitals throughout [Country). And on one end, the hospital has been almost completely paper based, with a separate computer system for pathology values, a separate one for discharge summaries, all the vital signs are recorded manually. All the blood gases are recorded manually. And those are all sort of electronic data storage apart from blocks of text. The other extreme has been, the hospital I’m working in that moment, which is just fully integrated. So, everything is all on one system and includes all the observations which are recorded manually by the nurses. So, there’s huge sort of data and variables available for where I am working at the moment. And it’s all integrated across the sort of lifespan of the person in the hospital.’ P8 ICU Physician HIC ‘Yeah. So, the online EMR that they use at [Hospital] it’s called [Software name), and that has everything in it, it’s like your bloods, medications, vital signs, everything is integrated into the one system. And even you’ll take a blood sugar, and it will automatically go across to the online system. Whereas [City] was pretty much all paper-based, all of the lab systems and everything were all segregated systems, and often things were then transcribed, the blood results will be transcribed onto paper. So, if you’re going to collect data about obs and things, you have to go around and individually, look at each patient.’ P28 ICU Nurse HIC
Secondary use of data		
Types	None	‘At the moment, no. So, 100% is for clinical care.’ P3 ICU Physician HIC ‘Not that I’ve seen. So mainly, it’s patient care, follow up of patients.’ P25 ICU Physician LMIC
	Quality benchmarks	‘In places where there’s manual data collection, it’s primarily been for benchmarking reports…But most ICUs, probably about 95% of ICUs in [Country] collect data that’s submitted to a central body that provides a benchmarking report. And so, that would be the only sort of routinely collected clinical data that’s sent off for benchmarking and reporting back. I’d say in the hospitals I’ve worked in, which had EMRs, or, you know, yeah, like databases, definitely it all got used for secondary purposes and quite frequently and quite a lot. And in places where it was manually extracted, then hardly ever.’ P8 ICU Physician HIC
	Research	‘The answer is yes. So, especially being a-, like an academic medical centre, we have like ongoing research all times within our critical care.’ P6 ICU Pharmacist HIC

HIC, high-income country ; ICU, intensive care unit; LMIC, lower-to-middle-income country.

### Views about using AI in ICUs

#### Perceived opportunities

Although there were large variations in knowledge of AI among participants, and the vast majority are currently not using AI technology in practice, all participants in both HICs and LMICs had a generally positive view of AI. Participants saw huge potential for the technology to be very helpful and improve patient outcomes in the ICU, although not all participants had a clear idea of what or how benefits would happen. Many participants, however, highlighted the potential benefits of AI in relation to their workload given the number of patients they needed to simultaneously look after and the impossibility of keeping track of all the information being constantly generated in the ICU. AI was seen as a tool to support intensive care professionals deal with this data overload and to do their jobs more effectively and efficiently; by providing an early warning system for patients deteriorating, predicting which patients are at greatest risk and reducing errors. Many participants also noted the potential for AI to improve workflows, such as helping to manage ICU bed capacity or improving the accuracy of documentation (table 3).

**Table 3:** Views about using AI in ICUs

Theme		
**Code**	**Subcode**	**Example quote**
Perceived opportunities		
Potential to improve outcomes		‘Yeah. So, I do think that the artificial intelligence is needed in the ICU. And I’ll tell you why. Because as a critical care physician, normally I have between ten and 12 patients that I have to take care. And then for me, it’s almost impossible to keep track all of all the information that is being generated every single minute in the ICU…Every single minute, what is happening with this patient. There are many, many, many little variations in the vital signs, many little variations in the dose of the medications. But is practically impossible for a human to keep track of all of that. And then when that’s in a 24 hours period then even more impossible…And sometimes in the ICU, we have this data overload. So, we really cannot handle. So, I do think that is important.’ P5 ICU Physician LMIC ‘I mean, I would say there’s definitely, definitely a huge, huge potential of improving patient, the outcomes by incorporating AI and patient data. And I mean, I can see it through some of the research that I’m doing. I can see it through some of the research that others are doing in the field of critical care in AI. There’s so much data. It’s probably one of the few fields that has so much data for individual patients and also for various patients.’ P7 ICU Pharmacist LMIC ‘I think it’s hugely useful. I think it’s going to potentially improve efficiency, improve outcomes, standardize management a lot more.’ P45 ICU Physician HIC
Concerns about use		
Validity	Bias	‘I think some things that people might worry about whether it’s representative, I guess, particularly in some of the communities that I have worked in, that whether AI is actually applicable to you know, indigenous people or people from different backgrounds. And I think that would provide some resistance to its uptake as well. And it would be a concern that I'd have as a clinician in terms of its validity and the people that I'm using it for.’ P8 ICU Physician HIC ‘If you have developed your predictive model on a subset of patients that is some and-, it’s somehow biased, it doesn’t reflect all patients. You know, there can be racial or biases, or all sorts of potential ways that your predictive model doesn’t apply to everybody. And so, but that’s just about getting the science right.’ P55 ICU Physician HIC
	Generalisability	
Explainability	Essential	‘What’s necessary in healthcare is explainable AI, we really need to know why the conclusion came up. Both for our own understanding for trust for the patient and the rest of the healthcare ecosystem, and also medical legal purposes, we need to know why the machine thought this was the right answer.’ P1 ICU Physician LMIC ‘Whenever people don’t understand how something works, it generates fear, it generate, you know, this feeling that they are not in charge. And that’s something for a critical care physician, you need to allow them to be in charge, you know. And in general in medicine, you don’t want to take away the decision capacity of the doctor. You don’t want to do that. You want to provide a tool, you know, that you are not replacing the doctor. Just making sure that they’re aware of the different alternatives. And at the end, they’re the ones making the shots.’ P5 ICU Physician LMIC ‘I think you should always understand exactly why you’re doing something especially when you’re working with people who are really, really unwell. If I always have to be able to explain why I’ve made a decision then probably the computer also should be able to explain why it’s made a decision.’ P28 ICU Nurse HIC
	Not a key concern if it works	‘Well, let’s put it this way. If the back box works…everybody’s going to be happy. People aren’t going to be happy once the black box stops working or once people use the back box for unintended cases.’ *P15 ICU Physician HIC* ‘I mean, implementation is a difficult subject because AI, the better AI gets and the more powerful AI gets, the less we’ll be able to understand why it comes to certain conclusions. And honestly, to me, this is actually one of the big benefits of AI, that AI can do things that we can't understand.’ P20 ICU Physician HIC ‘This is a hard one, because it depends. I think it depends more about the trust that people have in the in the in the technology. I think if you have evidence that the system works, even if it’s not explainable, I mean, I would personally be comfortable in using it. I think for most users, they would…I think it’s because I’m biased. I’m biased towards AI. I believe there was some studies looking at how you influence people using AI based on how much explainability they have, and people tended to request more explainability to trust their recommendations. I think as a general rule, it is probably important to try to provide explanations.’ P32 ICU Physician HIC ‘Coming from anesthesia, we have these black boxes. If you look at them for anesthesia monitoring, the precise algorithms are patented and we don’t have a clue. We just get a number. If you’re good, you can look at the raw EEG and sort of get an opinion whether the number is way off or the ballpark figure is correct. There are now monitors coming out looking at pain levels interoperatively. Same thing essentially, that’s a black box. Being an anesthetist, I don’t mind black boxes. I need to be aware when the black box could give wrong information. If you have a pain monitor that tells you everything is fine, and you’ve got a patient who’s hypertensive and tachycardic, is this a situation where the monitor might have a problem or is the patient actually in pain or is it just a hemodynamic problem and it’s not a pain issue? Being able to trust the machine to take these things apart would be extremely valuable. So I think the black box, if you’re used to using it, if you have a feeling for the limits, I’m not too reluctant to use something like that. As I said, we are used to that.’ P38 ICU Physician HIC ‘No, I don’t think so. A lot of clinicians aren’t data scientists and just don’t have the fundamental knowledge to be able to interpret an AI machine learning model. We could say a lot about the current methods we use, for example, of patient monitoring. I couldn’t accurately explain all the technology that goes into, for example, an arterial line. Yet I use the output for that and can understand fundamentally how it works. But I don’t necessarily understand the full physics and everything that goes behind it. I would say the same is with AI.’ P44 ICU Physician HIC ‘No, I don’t. I think if there was enough data to prove that it was safe, then I would say I don’t need to understand how it’s doing it for it to work. There’s so many things that we do at work that I don’t understand. I don’t really understand how a pulmonary artery catheter gives me data or how a dialysis machine does almost anything that it does. But I’m reassured by its safety profile and the rigorous processes of following up, its continuous safety monitoring. It wouldn’t concern me that I didn’t understand how it was doing what it’s doing.’ P45 ICU Physician HIC
Responsibility	Dependent on use	‘I think if you look at the AI tool as a tool that would function in place of a clinician then definitely liability would be an issue. But if you look at it as a tool, in addition to all of the other tools that helps in decision-making, and helps in patient management, then there should not be that liability issue. It’s kind of like saying, well, you know, you did the labs for the patient, and there’s a lab error. You as a clinician, you should look at the full picture and make that decision that this doesn’t match with the rest of the pieces that I have. So, I think again, if you look at it as that tool coming in and making that decision of saying, well, this patient has lung cancer or doesn’t have lung cancer, and then you start chemotherapy right away based on that machine then that’s where there is a liability issue that ideally, I think they should go hand in hand with the clinical, with the clinician’s decision or with the full assessment of the patient.’ P7 ICU Pharmacist LMIC ‘I think ultimately yeah, clinicians will have that accountability. So, it’s about how we would be applying AI just as it would be the same as how we would be applying any other technology that we use in intensive care…But I think yeah, I think ultimately, it’s still a technology. It’s not a sort of sentient being. So, ultimately, I think there yeah, in the intensivists will still be responsible for whatever happens.’ P8 ICU Physicians HIC
Dependency	Deskilling	‘First thing that popped in my mind is probably the recent deskilling. A lot of things, even from paper charts to electronic system, is a big step up…We made a lot of things very automated almost. So say, for example, on paper chart, the doctors would want us to have to write down exactly what amount of drug and in what diluent to put in and what the dose range would be. So by practice, they would then be familiar with it. Whereas right now, all they have to do is select the drop click, and everything is prebuilt on the system for them. Probably one of the worries I would get is if we take that tool away from them, then would they then struggle to then perform what they should have been doing in the first place?’ P33 ICU Pharmacist HIC ‘Yes. Drug calculation before everyone’s doing everything on paper, and I think if the system was down, I personally I think I forgotten how to do some of the like noradrenaline, how to calculate it, like quickly on the spot, like I don’t think we’re-, before it was something we would do it every single hour. So, it was basically drilled in your head. And now on the computer, suddenly, if the system is done, it’s like, what do I do now?’ P35 and P36 ICU Nurses HICs
	Dehumanisation	‘But I guess the other concern would be the dehumanization of it. So, I worry sometimes that the junior doctors are nowadays quite reliant on technology and looking at the screen rather than at the patient. And I think there is a risk of sort of going the other way. Forgetting that it’s the patient there.’ P3 ICU Physician HIC ‘Let’s see, I think the dehumanization? I don’t know if the term is clear, but that fear of being attended by machines, let’s say, people always expect the decision or the face to be given to them by someone else. And we believe that, I think that, if used properly, artificial intelligence is going to help us so that people can take care of those things that have to be done because we don’t have time, but people in general think that if we use artificial intelligence, it dehumanizes care. They distance themselves from the patient, and the patient feels that the caregivers are distancing themselves from the patient.’ P10 ICU Nurse LMIC ‘I think efficiency’s improved. It’s so easy to search for a keyword in someone’s medical record and find a specific entry from three months ago made by one person. You can access things from home or from your office without waiting physically for a file. As soon as patients come in through ED, you’ve got their history available. Lots of things like that. I think environmentally it’s, I presume it’s better, although I’m not actually sure what the environmental impact is of all the computers that are required to manage it. But certainly not wasting paper’s useful. I think there’s an efficiency in ordering a test and knowing that the receiver is going to get it immediately, and not waiting for a piece of paper to find its way down to radiology or something. Legibility of notes, and particularly medication charts I think has improved a lot. The things that I think have deteriorated, so I noticed junior medical staff and nursing staff spend so much time at the computer, often to the detriment of what’s actually happening with the patient. Particularly patients who are awake or not as ill, that really just need more of a personal touch. I think that the computer becomes partly a distraction, but also just a job that takes a lot of time. Which speaks to the inefficiency of the system, I guess, and I’m sure there are better systems than ours. But ours, the nursing staff spend a lot of time looking at the computer screen rather than the patient and the surroundings.’ P45 ICU Physician HIC
Disparity	Will widen gap between rich and poor	‘Of course, the richer or affluent places, they’re going to have more technology. They’re going to have the ability to implement these things. These poor county hospitals and predominantly rural black places in the US, they’re not going to be spending the money on that. They’re not going to have it. For sure there’s going to be disparities in the application and benefit from it. Until there’s, like at some point in 50 years, every hospital will have a basic amount of EHR and technology. Once you get to that everyone has a certain basic amount, then these tools will be everywhere. But that’s probably a long ways away.’ P39 ICU Physician HIC

AI, artificial intelligence; HIC, high-income country; ICU, intensive care unit; LMIC, lower-to-middle-income country.

#### Concerns about use

Most intensive care professionals, however, also held some well-known concerns about the use of AI in clinical practice. There were no important differences regarding the concerns expressed by participants from HICs and LMICs. Five key concerns emerged from the interviews:

##### Validity

A major concern raised by participants was regarding the risk of AI technology being biased and not generalisable. Participants were very concerned about AI applications not being applicable in real life to the majority of patients, particularly in ICU where there is such a heterogeneous group of patients. Participants were also concerned that AI technology would not work as well with minorities who are already disadvantaged (eg, Indigenous communities or those with limited healthcare access) if those groups are not sufficiently present in the training data set.

##### Explainability

Some participants thought explainable AI was necessary as they always needed to understand exactly why they were doing something when working with critically ill patients, and that a lack of understanding could generate fear and undermine the trust of clinicians and patients. However, most participants were not concerned about ‘blackbox’ AI applications and thought that evidence that an application was helpful and safe was far more important than explainability. These participants noted that they did not understand how many other technologies used in the ICU worked and that clinical judgement should not be based purely on an algorithm but should combine a range of patient information and professional expertise.

##### Responsibility

Most participants saw the issue of responsibility being dependent on how AI was used. If AI was used in place of a clinician, making changes to patient care independently, then the question of who would be responsible if things went wrong was seen as very problematic by many. However, if AI was used as just another tool to help clinical decision-making, then participants thought that there was no significant problem and responsibility would remain with the clinician.

##### Dependency

Many participants raised concerns about clinicians becoming too dependent and trusting of AI technology in the ICU and not using their own clinical judgement or skills. Participants saw this as part of a wider problem related to increasing digitalisation. Although this technology potentially has benefits, participants reported many junior staff becoming too reliant on technology, which was leading to (1) deskilling of staff, who can no longer do certain tasks themselves (eg, calculate dosages) because the system is down, and (2) a dehumanisation of care, with staff spending too much time looking at the computer screen to the detriment of personal care of the patient.

##### Disparity

Some participants were also concerned that there will be large disparities in the application and use of AI technology in ICUs, which is going to widen the gap between richer and poorer settings.

### Barriers and challenges to implementing AI in ICUs

Three overarching barriers to implementing AI in ICUs emerged (table 4):

1. Digital infrastructure: Participants from both HICs and LMICs identified the current digital infrastructure of institutions as a major barrier. Most participants reported that their institution has neither the technical capability (hardware and software) to capture the necessary data or run the algorithms, nor the staff with the right knowledge and skills to use the technology. Some participants in LMICs reported not even having a stable electricity supply. This pointed to ongoing structural problems in the organisation and delivery of healthcare, and many participants in both HICs and LMICs described how they worked in broken healthcare systems where funds were limited to varying degrees, and investing in digitalisation and AI is not a priority. They suggested that many decision-makers either did not understand the value of digital technologies for improving patient care or were too burdened by the existing financial strain on their health system.
2. Knowledge and understanding: Participants also identified a lack of knowledge and understanding about AI and the clinical context these tools will be implemented in as a significant barrier. Participants felt that this affected professionals’ and patients’ acceptance and willingness to use AI, and that the disconnect between clinicians and technical partners too often leads to non-optimal AI tools. Indeed, one participant described most AI applications as ‘solutions looking for problems’ that do not exist in the view of clinicians. Participants also reported that some colleagues’ views about data ownership and competition led them to be unwilling to share data, which was also reported to be a substantial challenge that undermines AI implementation.
3. Regulatory: Large variations in regulations regarding data protection within and across countries were also highlighted by participants as an important barrier. Some institutions and countries were reported to be significantly stricter than others with regard to data sharing and the secondary use of data. Although participants all agreed that protecting patient privacy was essential, they also felt that the current situation could potentially harm patients because it is undermining research and their ability to improve care.

**Table 4:** Barriers and challenges for implementing AI in ICUs

Theme		
**Code**	**Subcode**	**Example quote**
Digital infrastructure	Technical capability lacking	‘We don’t have the equipment. We need internet, we need trainings. We need to train people on that. I mean we need time to get used to it. At the same time, we need the computers in the department for that…But yeah, some of the things that would maybe delay implementing it will be like the equipment, maybe the orientation of staff on the equipment.’ P2 ICU Nurse LMIC ‘If you don’t have the infrastructure and the ability to gather the data and then run these algorithms on it, it’s going to be difficult. I think though that most of Western Europe and the US, and rich countries, are going to have, in the near future, fairly completely electronic systems. [Country] trying to get there, we're just bit far behind…Obviously that’s a barrier.’ P39 ICU Physician HIC ‘The main obstacle is actually the availability of a hardware and software together that can make things possible. The second obstacle is the people who will know how to use it, and to activate it.’ P43 ICU Physician HIC ‘In most low- and middle-income settings, I think this is, you know, when I’m working with my public health hat on access to high quality, reliable data is like the number one problem for public health research related, but also in this case, thinking about how to develop AI systems. And I think that is, without a question, the biggest challenge. Because it’s not just a problem of like the fact that we’re mainly working in paper-based records. But it’s also the fact that, you know, our electricity comes in and out. Our monitors when they stop working people don’t, you know, there are many, many layers to this to the point where we would be having reliable collection of data that can be used in this way. So, I think it’s-, that’s a challenge. And it’s not simply the fact that we don’t have an EHR to document data. There are many other components that feed into that.’ P50 ICU Physician LMIC ‘The thing is if we want to try to integrate artificial intelligence into my hospital in particular, we don’t have the technology so far. And the other big limitation, you know, is the electronic healthcare system. Because this piece of software is crazy expensive, and trying to integrate whatever you are generated from data perspective, that could be challenging….Yeah. So, what is missing is a way that the data is transferred directly from the ventilator to the electronic healthcare system. And for doing that, you need a piece of hardware and software that allows you to extract the data from the ventilator and put it in the chart. And the thing is, in Colombia, we have many different makers from for the ventilators, for the fusion bombs, all of that sometimes is tough…’ P5 ICU Physician LMIC ‘But the biggest problem is the missing digitalization of the data, a lot of also University Hospitals in [Country] are working on paper, so they have no data available on a server. That’s the biggest problem.(Question: Could you estimate how many hospitals would have currently the possibility of using AI technology?)Probably 20–25% of the hospitals actually…But I think this will change in the next five years, probably. We’ll arrive probably to 50 or 60% of the hospitals who are used then have all the data available in digital form. But it depend also from the politicians.’ P14 ICU Physician HIC ‘We probably haven’t had the bandwidth to think about the other things. Because as you say, if you don’t have digital data the rest is irrelevant. I would love to have digital data…But you know, let’s start with some basic stuff first, which is a monitor that doesn’t have to be turned into a paper record by a nurse every hour. I guess the advantage of paper is, you know that it’s secure. It’s physically in one place. It can’t be accessed by anyone else. But you know, the disadvantage to that is it’s secure and can’t be accessed by anyone else. So, you certainly can’t do analysis on it. Yeah, I think we’re a long way behind.’ P47 ICU Physician HIC
	Staff with right knowledge and skills lacking	
	Insufficient funding available	‘The [Country] system is, the geography’s huge. The patients they have to service, the land mass is huge. They haven’t updated and put enough money into our system. We have two year waits for hip replacements, two year waits for cataract surgery, chronically underfunded. We’ve had aging population with mass migration on top of that…Our system is so broken that the electrification or EHR computerization of our healthcare system was not a high priority in the funding list. Some provinces have been more aggressive, the wealthier provinces…I think [Country), and certainly the less affluent parts of [Country] that don’t have this basic infrastructure, AI is not a priority. There’s no money going into that at all.’ P39 ICU Physician HIC ‘So, the first thing is that we don’t have enough money to implement any additional systems other than increasing capacity to provide critical care. And it’s not to say that all these things are considered a nice to have. But if you gave me a million dollars, and said, you can either spend this on an AI learning system, or you can open another two ICU beds, I know what I’m going to choose, and it’s not the AI learning system. So, I think the biggest barrier we currently have is simply funding.’ P47 ICU Physician HIC ‘I would say that the biggest barrier in South American in general. Because for you to be able to collect everything into the dataset, then you need to have a piece of software that can extract data, transfer that to the system. And that for doing that, you need to invest money. You can imagine ICUs in South America, they don’t have money to buy ventilators. Of course, they prefer not to invest money in a piece of software that they don’t see how it can affect the patient care.’ P5 ICU Physician LMIC
Knowledge and understanding	Disconnect between clinicians and technical partners	‘There are times where I wish as a clinician, I had the abilities to do everything myself, including like, data extrapolation, writing my own data. Because sometimes I feel like there is sometimes-, not a disconnect, but lack of connection between your data analyst or data scientist that helps you develop your project or helps you, like that’s their project, and you’re trying to help them that the-, my lack of understanding of artificial intelligence vs their lack of knowledge from clinician perspective, which is understandable for both parties, leads to sometimes like two sides not understanding each other as well as they should be.’ P6 ICU Pharmacist HIC
	Insufficient focus on what is needed	‘I think it would be good to see what people really want from AI. Instead of saying, okay, here’s the new technology and here’s what it can do. Maybe we need to think about, what is it that we’re lacking in our current practice that we feel we need? And then see if AI can quickly fill that void. That may be a better way to push AI forward and gain acceptance. Rather than saying: “Hey, look, here’s AI, it can do all these things.’” P3 ICU Physician HIC ‘So the idea that I have, we know the problems. IT doesn’t know-, they don’t know what problems we are suffering. So, the IT, they providing solutions for a problem, which are most of the time not existing in our eyes. So, this disconnection that makes things with doesn’t match. Why you’re advertising? Why you are selling me this machine? I don’t need it.’ P43 ICU Physician HIC
	Views or data ownership and competition	‘Most of them don’t share it because of commercial aspects and secrets. I have a lot of anger, it’s not ethical, it’s not their data, and…I think that it’s not a commercial aspect, it’s the ego aspect. They want to publish papers in the New England Journal or whatever. They want all the data.’ P52 ICU Physician HIC ‘I would say both. I would say data protection laws, and it’s political. Competition between academic centers. We have the bigger data set. So if we open it, people are going to publish using our data. Very classical.’ P32 ICU Physician HIC
Regulatory	Large variations in data protection	‘It’s not too much of a major issue in [Country). If it’s data that I’m pulling from my own institution, it’s de-identified and I’m using it for research purpose. I think the issue becomes when you start doing multicenter studies and how do you kind of pull data from different institutions, and then it goes into one pool. There are some institutions that are more strict than others, and some countries that are more strict than others. So, I think this is where the difficulties come into. And I think countries and institutions are realizing that they need to be less strict about those criteria. Because, yes, we are protecting the privacy of our patients by having all these measures. But then at the same time, there’s potential harm if you’re doing all this to these restrictions, that there’s no-, or there’s minimal research that people are doing. So, you don’t understand your patients fully. You don’t give them the full care. You don’t have research. So, it’s, I mean, it’s you have to kind of weigh things both ways. I’m not saying that you just kind of you know, open things and have all the data freely available. But at the same time, too many unnecessary restrictions, I think makes it difficult to conduct research, and then that has its own issues there. So, it’s kind of a balance between both.’ P7 ICU Pharmacist LMIC **‘**Yeah, I think it’s a huge issue. Again, there’s huge regional variation. So, in some states all the hospitals have a shared system, and you add a baseline to set a clinical level, you’ve got access to all the other hospital information. So, it makes things like linking data very easy. Because it is already linked within the state. The state that I’m in does not do that. And so, it’s all separate, which already just makes it like practically very difficult. Let alone, you know, having to deal with de novo sort of ethics, submissions, and trying to actually get that all together.’ P8 ICU Physician HIC

AI, artificial intelligence; HIC, high-income country; ICU, intensive care unit; LMICs, lower-to-middle-income countries.

### Facilitators for implementing AI in ICUs

Three key suggestions for facilitating and improving the implementation of AI in ICUs emerged (table 5):

#### Demonstrating the value/limits of AI

**Table 5:** Facilitators for implementing AI in ICUs

Theme		
**Code**	**Subcode**	**Example quote**
Demonstrating the value/limits of AI	Evidence of AI utility and reliability	‘I think some strong studies will help of course, if you can show that AI helps tremendously with prediction of mortality or prediction of hypotension or with mechanical ventilation with better outcomes. And those results are constantly produced and coming from different countries, then I think it will be difficult for a doctor to ignore it.’ P27 ICU Physician HIC ‘I think more randomized control trials should be done to prove that these technology can really improve the patient’s outcome, not just to choosing a model, but to see whether the model, the usefulness of the model, to improve reduce mortality or reduce costs. If we can prove least in the large RCTs, this will be a very important indication.’ P29 ICU Physician LMIC ‘I think one thing that will help is very, very solid research. And not only by people wearing pink glasses and saying, “Oh it’s so good.” But really solid research. I think, given the current way we practice medicine, I think that’s the best way to establish new techniques. The whole technical side. Well, people are all enthusiastic about it, that will happen. But really validating stuff and also making clear that once validated…What do we need? For instance, how many times do you need to update a model? All these kinds of things are below the surface, whereas I think they’re extremely important.’ P31 ICU Physician HIC ‘I think you’d have to prove its value. So whether that’s making life easier for clinicians or making outcomes better for patients. I think you’d have to show that there’s a value in it.’ P45 ICU Physician HIC
	Clear explanation of strengths/weaknesses and advantages/disadvantages of	‘As far as implementation goes, I think there has to be a clean explanation of the strengths and weaknesses and disadvantages of the software, that this is not like a God to predict everything for you. Like you still have to use your clinical knowledge and your brain basically, at the end of the day before making a decision. And don’t just say purely, well because what the algorithm told me I have to do this. It’s really how to utilize the algorithms rather than abusing them for the purposes of clinical decision-making processes.’ P6 ICU Pharmacist HIC
Closing the gap of understanding	Training and education	‘I think it’s very important to start early, and they need to be primers on data science, machine learning and AI, right? From the med school level now, start educating people on what it’s all about.’ P1 ICU Physician LMIC ‘The way I see AI it’s like a new discipline within medicine, for example. I as an ICU doctor, I will not go to the lab and challenge your potassium result. Right? So, I rely on the lab, making sure that that potassium result is correct. Measuring potassium is very difficult, like it’s not an easy thing, you have to think about hemolysis, you have to think about the quality of the blood, you have to think about whether the blood has been standing long enough. You have to think about whether your reagents were correct, whether the controls were correct, whether your machine is competing correctly, whether it was the same, the right patient. So, there are so many things that we take for granted anyway, in reading a simple potassium result. So, I don’t think that would be one of the things that I really concern me. So, I would not say: “Okay, I’m not going to use AI unless I understand all the algorithms.” I don’t think that would be the case, but certainly, I think a new discipline. So, for example, like a doctor trained in AI, who’s working in the hospital with good confidence of other teams like about critical care physician to use it. So I would see, the way forward would be a new specialty within medicine, where they have specialist trained in artificial intelligence, who are equipped with the skills required to make valid predictions, make valid algorithms, and then I’ll trust that person, rather than me trying to learn through it again, which would be impossible.’ P3 ICU Physician HIC ‘I would say education is a large part of it, you know, getting people, not just clinicians, both data scientists, IT and clinician, everyone, to kind of understand the concept more, see the value of it, see what it means. And how do I actually apply it? How do I develop models? How can I incorporate that? And then that would get people a bit outside their comfort zone so that they can kind of take on the next steps sort of. I would say education would be a big part of it.’ P7 ICU Pharmacist LMIC ‘I think it needs to become part of the curriculum that we teach critical care clinicians. I think clinical informatics in some way needs to be taught. And how, what we don’t get taught is we use a lot of these IT systems and electronic medical records as clinicians, but we don’t ever get taught around the backend. How do we use it? How do we actually use all this stuff that we put in and this data that we aggregate, but we barely touch. So, what, so for me, it’s about gradually teaching people how to do it. And then as a result, if you do have these large data sets, what’s the way of working with some expert in a local area or in a collaborative group to work on a project that develops something. And so, they’re that sort of, they’re the things that would in my mind, help people become, understand the issues, but also build a skill set that helps the next generation do it better.’ P19 ICU Physician HIC
	More inclusion of clinicians	‘And also, have discussions with people who develop these things so that they can interface and interact with doctors and nurses, and they don’t build something which won’t get used. So, a lot of the apps that are built are crazy good in terms of the technology. But no doctors were asked what they wanted.’ P1 ICU Physician LMIC ‘It should be designed by the people who actually will use it and will need it. I think it cannot be designed by anybody who’s not familiar with the processes…you need the collaboration between the end user and the programmer at the start.’ P40 ICU Physician LMIC ‘This connection between the physicians and the IT guys. This is the most important thing. We have disconnected.’ P43 ICU Physician HIC ‘Additionally, it ultimately depends on who builds the model that is used. But I feel like clinicians definitely should be involved.’ P9 ICU Pharmacist HIC

AI, artificial intelligence; HIC, high-income country; ICU, intensive care unit; LMICs, lower-to-middle-income countries .

Participants thought that clear and consistent evidence from robust research studies confirming the utility and reliability of AI applications would be the most important facilitator for increasing the acceptance of and willingness to use AI applications in ICUs. Participants also saw a need for a clear explanation of the strengths/weaknesses and advantages/disadvantages of each application.

#### Closing the gap of understanding

Participants made two main suggestions for improving the current gap of understanding between clinicians and technical partners, to increase the acceptance and the clinical utility of AI in ICUs:

- *Training and education:* Many participants noted the need to improve training and education of both clinicians and data scientists, so clinicians have a better understanding of AI concepts and data scientists understand the clinical context better. Although participants saw the need to improve all intensive care professionals’ knowledge and skills in this area, some participants also advocated for a new (sub)specialty where clinicians are trained in AI as it was unrealistic to think that all clinicians could be trained to the required level.
- *More inclusion of clinicians:* Participants strongly felt that there needed to be more consultation and involvement of clinicians from the beginning in the design and development of AI applications for ICUs, to improve the connection between clinicians and developers and the resulting product.

#### Improving ecosystems

Participants also saw the need to improve the wider ecosystem, including: ensuring that there is a proper system of data collection and documentation, that funding bodies are aware of bottlenecks so funding is directed to efforts to translate research into practice rather than just generating more accurate prediction models, that grant panels have the right expertise to evaluate multidisciplinary research and enhance the potential for academic/commercial partnerships.

## Discussion

This is one of the largest qualitative studies to date to examine the views of intensive care professionals regarding the use and implementation of AI technologies in ICUs, involving 59 participants from 24 countries, including countries from Europe, Asia, North America, South America, Middle East, Australasia and sub-Saharan Africa. This study found general agreement among participants’ views regarding the use and implementation of AI in ICUs, which were largely in line with existing empirical research with ICU professionals.^13 14 19 20^ Participants had generally positive views about the potential use of AI in ICUs but identified important technical and non-technical barriers to the implementation of AI. A key finding of this study, however, was important differences between ICUs regarding their current readiness to implement AI. It was striking that these differences were not primarily between HICs and LMICs as might be expected. Rather, the key difference was between a small number of ICUs in large tertiary hospitals in HICs, which were reported to have the necessary digital infrastructure for AI, and nearly all other ICUs in both HICs and LMICs, which were reported to neither have the technical capability to capture the necessary data or run AI algorithms, nor the staff with the right knowledge and skills to use the technology. Although technical barriers to implementing AI in ICUs have been widely discussed,^4–6 11 14^ intensive care medicine needs to be careful not to gloss over the importance of the current readiness of ICUs to implement and use AI, otherwise it will risk building a house of cards. Pouring massive amounts of resources into developing AI without first (or in parallel) building the necessary digital and knowledge infrastructure foundation needed for AI is unethical.^32^ We do not see the possibility of real-world implementation and routine use of AI in the vast majority of ICUs in both HICs and LMICs included in our study any time soon, and we do not think this ‘last mile’ of implementation^33^ will be reached unless the necessary digital and knowledge infrastructures are built first. We are of the view that ICUs should not be using AI until certain preconditions are met. Intensive care societies from around the world need to come together and reach a consensus on what these preconditions should be.

### Limitations

This is a qualitative study that did not collect statistically representative data. However, we included a range of intensive care professionals from 24 HICs and LMICs, which makes it likely that this study has captured key aspects of a multisided issue. A bias might exist toward the reporting of socially desirable attitudes,^34^ however, given our results that are rather critical of current practice, we believe that such a bias is limited. The study was carried out across 24 countries, and there may be some regional and country-specific differences that might limit the generalisability. Nevertheless, many of the key issues are associated with aspects that are common in all countries (eg, limited digital data collection and documentation, and an underutilisation of patient data in ICUs), these findings are likely to be of wider international interest. There is currently no established definition of what constitutes AI, and a definition of AI in medicine was not provided to participants. As noted in the results section there were large variations in knowledge of AI among participants, and concrete examples were provided where needed. However, this may have affected the ability of some participants with limited knowledge of AI to answer some questions. The study was also undertaken before the explosion of interest in the use of LLMs and the chatbots that they power. The AI discussed in this manuscript therefore does not include LLMs.

## Data Availability

Data are available upon reasonable request. Our data include pseudonymised transcripts of interviews, which cannot be made publicly available in their entirety because of (1) the terms of our ethics approval; and (2) because participants could be identifiable if placed in the context of the entire transcript. This is in line with current ethical expectations for qualitative interview research. We provide anonymised quotes within the paper to illustrate our findings (corresponding to transcript excerpts), and the complete interview guide used in the study has been included as a Supplementary Information.
