# Supplementary material for: Building a house without foundations? A 24-country qualitative interview study on artificial intelligence in intensive care medicine
Source: BMJ Health Care Inform. 2024 Apr 19;31(1):e101052. doi: 10.1136/bmjhci-2024-101052 (PMC11033632; doi:10.1136/bmjhci-2024-101052)
Supplement: Supplementary data [file bmjhci-2024-101052supp001.pdf]

Supplemental Text 1: Extended Methods

The methods of the study are presented in accordance with the "Consolidated criteria for reporting qualitative research" (COREQ) [1].

Research team and reflexivity

Personal characteristics: Interviews were primarily conducted in English by S.M., a male senior researcher in biomedical ethics. Seven interviews were conducted in Spanish by A.F., a female medical anthropologist. Both interviewers have longstanding experience with qualitative research.

Relationship with participants: No relationship was established between the interviewers and the participants prior to the study and participants received limited information about the interviewers. There was no hierarchical relationship between the interviewers and the study participants.

Study design

Theoretical framework: The theoretical framework employed in this study was conventional content analysis [2].

Participant selection: Critical care professionals were primarily selected through purposive sampling [3]; to ensure that participants were from different backgrounds. Additional participants were identified using snowball sampling [4]. Participants were contacted by email and provided information about the study design and aims and rights as participants. Suitable dates for an interview were found with those willing to participate. Verbal consent was obtained from all participants directly before the interview and audio recorded. A total of 59 critical care professionals from twenty-four countries agreed to participate in the study and were recruited (see **Table 1**).

Setting: Interviews were held between December 2021 and August 2022. All interviews were conducted via a telephone or video call in English, except for 7 interviews which were held in Spanish. Only the participant and the researcher were present during the interview. Overall, 66.1% (39/59) of stakeholders were male presenting, and 33.9% (20/59) were female presenting.

Data collection: A researcher-developed semi-structured interview guide was developed to guide the discussion (see **Supplementary Information 2**). Based on the first two interviews that did not show any problems, it was decided that no further piloting or adaptation of the interview guides was necessary. No repeat interviews were carried out. Interviews were audio

recorded, no field notes were taken. Interviews lasted an average of 21 minutes (range 12–55 minutes). The interviews were transcribed in full and checked for accuracy. After 59 interviews, the question about data saturation arose and it was concluded that saturation was reached in the content and attitudes expressed by the participants [5]. Transcriptions of the interviews were returned to all participants with an invitation for them to review the transcription and send any corrections or clarifications; one response was received with minor corrections to syntax.

Analysis and findings

Using the interview transcriptions in their original language, S.M. performed conventional content analysis with the assistance of the qualitative software MAXQDA v11. Analysis commenced after interviews were completed. Initial themes identified common across participants as well as those unique to individuals were labelled using a process of open coding. Findings are presented as higher- and lower-level categories. The other investigators [A.F., L.A.C.] reviewed the initial analysis to clarify and refine codes, and conversations among the investigators continued until coding differences were resolved and consensus was achieved.

Ethics Approval

This study received approval (621/21 S) from the Technical University of Munich´s Research Ethics Committee on 23 November 2021.

References

1. Tong A, Sainsbury P, Craig J. Consolidated criteria for reporting qualitative research (COREQ): a 32-item checklist for interviews and focus groups. Int J Qual Health Care. 2007;19(6):349-357. doi:10.1093/intqhc/mzm042
2. Hsieh HF, Shannon SE. Three approaches to qualitative content analysis. Qual Health Res. 2005;15(9):1277-1288. doi:10.1177/1049732305276687
3. Palinkas LA, Horwitz SM, Green CA, Wisdom JP, Duan N, Hoagwood K. Purposeful sampling for qualitative data collection and analysis in mixed method implementation research. Adm Policy Ment Health. 2015;42(5):533-544. doi:10.1007/s10488-013-0528-y
4. Marshall MN. Sampling for qualitative research. Fam Pract. 1996;13(6):522-525. doi:10.1093/fampra/13.6.522

5. Fusch PI, Ness LR. Are we there yet? data saturation in qualitative research. Qual Rep. 2015;20(9):1408-1416. doi:10.46743/2160-3715/2015.2281
