# Supplementary material for: Building a house without foundations? A 24-country qualitative interview study on artificial intelligence in intensive care medicine
Source: BMJ Health Care Inform. 2024 Apr 19;31(1):e101052. doi: 10.1136/bmjhci-2024-101052 (PMC11033632; doi:10.1136/bmjhci-2024-101052)
Supplement: Supplementary data [file bmjhci-2024-101052supp002.pdf]

Interview guide

Introduction

Thank you again for agreeing to speak with me.

As described in the study information sheet, we are currently conducting a project funded by the NIH to explore how artificial intelligence can be ethically implemented in critical care medicine.

As a someone working in a critical care center, we are interested in talking to you today about your views and experiences regarding the use of data science and artificial intelligence in critical care medicine.

I will be audio-recording the interview so that I don't have to take notes, but transcripts will be de-identified so you or your institution cannot be identified.

Do you have any questions before I start the recorder?

Interview Questions

1. To start, could you say a little about your role in critical care?

2. Could you tell me about how the following patient data are collected and documented in your ICU: vital signs, medications, input and output, laboratory tests, progress notes, diagnoses, procedures?
   a. Are these data are being used for purposes other than patient care, e.g. generation of reports, research?
   b. What legal basis was used (consent, anonymization, exemption)

3. What do you think about the use artificial intelligence technology in critical care medicine? Do you think it is needed and will be helpful? How do you think it will affect your work and do you have any concerns regarding its implementation?
   a. Explainable
   b. Accountability/liability
   c. Bias

4. What do you think are the key barriers and challenges to ethically implementing artificial intelligence in critical care medicine?

5. What would you suggest to facilitate/improve the ethical implementation of artificial intelligence in critical care medicine?
